# Rare Combination of Talar Body and Bimalleolar Fractures: A Case Report

**DOI:** 10.3390/reports9010071

**Published:** 2026-02-27

**Authors:** Alexandros Tsioupros, Constantinos Chaniotakis, Konstantinos Zampetakis, Panagiotis Ioannou, Ioannis Ktistakis

**Affiliations:** Department of Orthopaedics and Traumatology, “Venizeleion” General Hospital of Heraklion, 44 Leoforos Knossou, 71409 Heraklion, Crete, Greece; alexandros-tsioupros@hotmail.com (A.T.); k_zampetakis@yahoo.com (K.Z.); panayiotis.ioa@gmail.com (P.I.); giannis.ktistakis@gmail.com (I.K.)

**Keywords:** talus fracture, bimalleolar fracture, ankle fracture, foot injuries

## Abstract

**Background and Clinical Significance**: Talar body fractures are very rare injuries, and their occurrence alongside ipsilateral fractures is even more uncommon. We present a case of a 40-year-old male who sustained a talar body fracture combined with an ipsilateral bimalleolar fracture after falling from a height, a combination previously described in only two cases. **Case Presentation**: Open reduction and internal fixation (ORIF) were performed using dual approaches for both the talus and malleolar fractures. Postoperatively, the rehabilitation protocol included a non-weight-bearing short leg cast, followed by partial weight-bearing with a controlled ankle movement (CAM) boot. At one-year follow-up, the patient achieved an American Orthopedic Foot and Ankle Society (AOFAS) score of 90 and reported minimal pain. Radiographs demonstrated minimal osteoarthritic changes and no signs of osteonecrosis. Nevertheless, early signs of osteonecrosis (ARCO grade I) were detected on MRI 15 months postoperatively. **Conclusions**: This case highlights the rarity of such injuries, outlines our institution’s treatment approach, and emphasizes the importance of long-term follow-up to monitor for complications such as post-traumatic arthritis and osteonecrosis.

## 1. Introduction and Clinical Significance

Talar fractures are considered uncommon in routine clinical practice, but they are the second most frequent tarsal bone fractures after those of the calcaneus, with an incidence ranging from 0.1% to 0.85% of all fractures [1]. The most common talar fractures are talar neck fractures with a reported neck-to-body ratio of 6:1. Talar body fractures are rarer and account for 13–23% of talar fractures. These fractures usually result from axial compression of the talus between the tibia and calcaneus [2]. These fractures are particularly significant due to their potential for severe complications, including avascular necrosis and post-traumatic arthritis [3]. It has been shown that a higher risk for avascular necrosis is associated with more displaced fractures and talar neck fractures as well as open injuries [4]. Even more rarely, talus fractures are accompanied by additional ipsilateral fractures. This report presents an extremely rare case involving a talar body fracture with an ipsilateral bimalleolar fracture. Only two such cases have been reported in the literature [5,6].

## 2. Case Presentation

**Figure 1:**
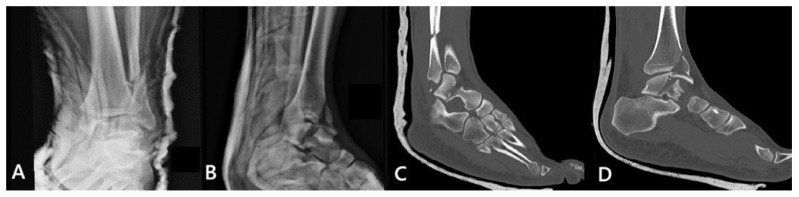
(**A**) Anteroposterior and (**B**) lateral preoperative X-ray views of the ankle, showing a talar body fracture combined with an ipsilateral bimalleolar fracture. Sagittal planes in the CT scan of the ankle showing the talar body fracture along with (**C**) the lateral malleolus fracture and (**D**) the medial malleolus fracture, respectively.

A 40-year-old male was admitted at the emergency department (ED) after a fall from a height of 4 m. Upon presentation, there was noticeable swelling and a clear deformity of the left ankle. Routine imaging was performed with X-rays of the affected limb at first, which revealed a closed left talar body fracture combined with an ipsilateral bimalleolar fracture (Figure 1A,B). The patient was neurovascularly intact, had no additional injuries, and had no significant past medical history. A short leg cast was applied at the ED for comfort and support, followed by a computed tomography (CT) scan for preoperative planning (Figure 1C,D). The talar body fracture in this case could be classified as a shear fracture in the coronal plane with an approximate displacement of 8 mm. Hence, the injury could be classified as type B according to Sneppen [3]. The combined ankle fracture consisted of a transverse medial malleolus fracture and a lateral malleolus fracture classified as Danis–Weber type C. The subtalar joint remained congruent and no osteochondral lesions or talar dome impaction were identified.

**Figure 2:**
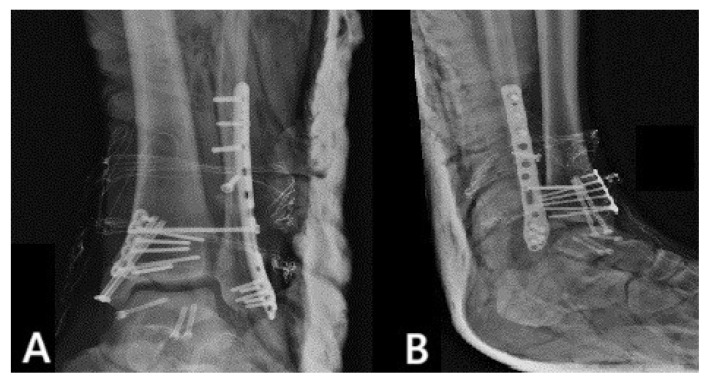
(**A**) Anteroposterior and (**B**) lateral immediate postoperative X-ray views of the ankle showing ORIF of the bimalleolar fracture and ORIF of the talar body fracture.

The patient was transported to the operating room (OR) for definitive treatment ten days later, once the swelling had subsided and the soft tissues had improved. One gram of cefoxitin was administered within 30 min prior to incision and discontinued 24 h postoperatively. For the fixation of the talus fracture, dual anteromedial and anterolateral approaches were employed. The rationale behind this selection was to permit direct visualization of both fracture planes, ensure more accurate reduction in articular surfaces and assist in the preservation of the blood supply. The choice of dual incisions instead of a single extensile approach may help limit soft tissue detachment and periosteal stripping, thereby preserving blood supply. Care was taken to preserve all soft-tissue attachments to the talar body as much as possible. Particular attention was paid to avoid injury to the deltoid artery branches, which arise posteromedially and supply the medial two-thirds of the talar body. Intraoperatively, the joystick manipulation technique was used as a reduction method, by inserting k-wires into fractured bone fragments. Three 3 mm headless compression screws were then inserted: two anterior-to-posterior and one anteromedial-to-posterolateral. Subsequently, medial malleolus was fixed through an extension of the anteromedial approach using two 4 mm cannulated screws along with a plate. Lastly, open reduction and internal fixation (ORIF) of the lateral malleolus was performed through a lateral approach, using an anatomic plate. Ankle stability and the syndesmotic complex were assessed intraoperatively by performing stress radiographs, and a syndesmotic screw was inserted. Fluoroscopy was used intraoperatively throughout the procedure to assess fracture reduction and ensure correct hardware positioning. Figure 2 shows the osteosynthesis of the fractures in the immediate postoperative X-ray views.

The postoperative protocol included a non-weight-bearing short leg cast for the first six weeks, followed by another six weeks of partial weight-bearing with a controlled ankle movement (CAM) boot and physiotherapy. The patient received low-molecular-weight heparin (LMWH) prophylaxis throughout his hospitalization and for a total of 6 weeks postoperatively to prevent deep vein thrombosis and pulmonary embolism.

**Figure 3:**
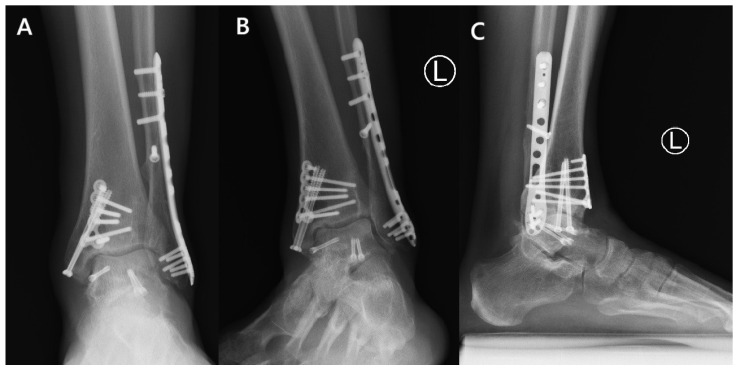
(**A**) Anteroposterior, (**B**) mortise, and (**C**) lateral weight-bearing X-ray views of the ankle at the final follow-up, showing fracture healing without complications but with minor post-traumatic changes.

At follow-up, X-rays were performed and the American Orthopedic Foot and Ankle Society (AOFAS) hindfoot score was used to evaluate the patient, assessing both functional outcomes and pain. The patient was monitored for a total of twelve months, achieving scores of 87 at six months and 90 at one year postoperatively. No limitations to his daily activities were reported. Slight pain on weight-bearing was reported only when walking continuously for a distance of about 5 km. Follow-up ankle X-rays indicated minimal osteoarthritic changes. No crescent sign or other signs of osteonecrosis or talar dome collapse were observed radiographically during follow-ups (Figure 3).

**Figure 4:**
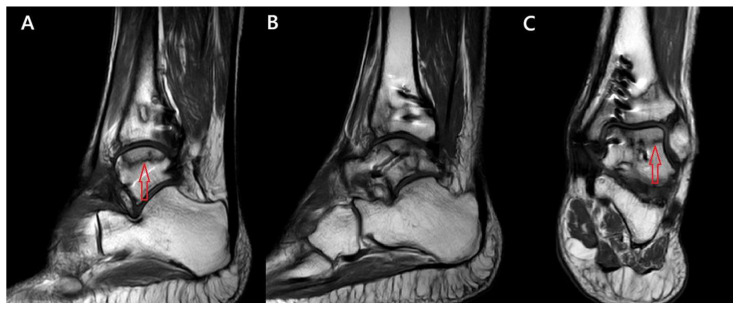
(**A**,**B**) Sagittal and (**C**) coronal MRI planes, revealing the early signs of talar AVN. The red arrows indicate the regions of AVN of the talus.

Despite the fact that the patient remained asymptomatic and radiographic imaging showed no evidence of avascular necrosis, advanced imaging with Magnetic Resonance Imaging (MRI) at 15 months postoperatively was performed, revealing possible early signs of the condition. Early signs of osteonecrosis were observed in the lateral two-thirds of the talar dome, classified as Association Research Circulation Osseous (ARCO) classification grade I (Figure 4).

## 3. Discussion

Talar fractures are rare injuries and the current standard treatment for displaced fractures of the talar neck and body is open reduction and internal fixation to ensure anatomic reduction [1]. They are associated with devastating complications such as post-traumatic arthrosis of the talocrural and subtalar joints, avascular necrosis (AVN), and malunion [3]. Talar neck fractures have been reported to have a higher risk of developing AVN (47%) compared to talar body fractures (26%) [4]. Follow-up assessment of our patient produced satisfying results. Lindvall et al. assessed patients with displaced talar fractures. Regarding post-traumatic arthritis, they reported an average AOFAS hindfoot score of 63.2 points for patients with the involvement of the subtalar joint alone and 60.4 points for those with combined subtalar and ankle post-traumatic arthritis. Regarding osteonecrosis, the average AOFAS hindfoot score reported was 49.5 points in the group in which osteonecrosis developed and 72.9 points in the group without. Nevertheless, Lindvall et al. included patients with a minimum follow-up of 48 months [7]. This highlights the potential devastating progress of these injuries. Adding to this a bimalleolar fracture, the outcome could be disastrous. In our case, the short-term functional outcome is better compared to Lindvall’s results. On the other hand, taking into consideration the shorter follow-up and the early signs of osteonecrosis, we conclude that further follow-up is essential.

Very few cases have been reported in the literature describing talar fractures combined with ipsilateral ankle fractures (Table 1). A literature search was conducted using PubMed to identify reports of talus fractures associated with ankle fractures. The following keywords were used in various combinations: “talus,” “talar body,” “talar fracture,” “ankle fracture,” “bimalleolar fracture,” and “malleolar fracture,” using Boolean operators (AND/OR). Table 1 summarizes the results of our literature search and underscores the distinctiveness of our case. To the best of our knowledge, this is only the third reported case of a talar body fracture occurring in conjunction with a bimalleolar fracture [5,6].

## 4. Conclusions

This is a case report of an extremely rare combination of fractures, and we present our institution’s approach to the management of such an injury. Considering the disabling nature of this type of injury, the associated high rates of chronic pain, and the difficulties during rehabilitation [3], our patient produced satisfying short-term functional outcomes following our treatment strategy. However, radiographic–clinical dissociation was observed and further follow-up and long-term surveillance is of paramount importance given the progressive nature of avascular necrosis and its potential consequences.

## Figures and Tables

**Table 1:** Combined talus fracture with malleolar fractures.

Injury Pattern	Published Case Reports
Talar neck fracture + bimalleolar fracture	Chen et al. [8] Radaideh et al. [9] Jadib et al. [10] Montane and Zych [11]
Talus fracture + medial malleolus fracture	Oesman and Nugroho [12] Zhang et al. [13] Arkesh et al. [14] Milenkovic and Stanojkovic [15] Mechchat et al. [16] Clement et al. [17]
Talar body fracture + trimalleolar fracture	Kar et al. [18]
Talar body fracture + bimalleolar fracture (same pattern as present case)	Verettas et al. [6] Elibrahimi et al., *Springer* case report [5]

## Data Availability

The original data presented in the study are included in the article, further inquiries can be directed to the corresponding author.

## Abbreviations

The following abbreviations are used in this manuscript:

CAM	Controlled ankle movement
AOFAS	American Orthopedic Foot and Ankle Society
ED	Emergency department
CT	Computed tomography
OR	Operating room
ORIF	Open reduction and internal fixation
LMWH	Low-molecular-weight heparin
MRI	Magnetic Resonance Imaging
ARCO	Association Research Circulation Osseous
AVN	Avascular necrosis
